# Assessing moral injury in health care workers living in secular societies: Introducing the Health care‐Moral Injury Scale (HMIS)

**DOI:** 10.1111/bjhp.12810

**Published:** 2025-06-12

**Authors:** Kathryn Fradley, Fuschia Sirois, Richard Bentall, Jaydip Ray, Rhian Bishop, Jonathan Wadsley, Sarah Danson

**Affiliations:** ^1^ School of Nursing and Public Health Manchester Metropolitan University Manchester UK; ^2^ Department of Psychology Durham University Durham UK; ^3^ Department of Psychology University of Sheffield Sheffield UK; ^4^ Department of Neuroscience University of Sheffield Sheffield UK; ^5^ Sheffield Teaching Hospital NHS Trust Sheffield UK; ^6^ Department of Oncology and Metabolism University of Sheffield Sheffield UK

**Keywords:** health care workers, mental health, moral injury, psychometrics

## Abstract

**Purpose:**

The adverse impact of moral injury on health care workers is well documented, for example during the COVID‐19 pandemic. However, currently available measures are unsuitable for assessing moral injury in health care workers living in secular societies such as the United Kingdom. The current study introduces and validates the Health care‐Moral Injury Scale (HMIS).

**Method:**

The 10‐item HMIS was designed during the COVID‐19 pandemic to assess moral injury in health care workers. Between September and October 2020, 858 health care workers completed the scale and other measures. A factor structure was identified by exploratory and confirmatory factor analysis and correlations were used to test the convergent (burnout, hope, mistrust) and divergent (loneliness) validity of the HMIS. Regression analyses tested the criterion‐related validity of the HMIS against measures of depression, anxiety and PTSD.

**Results and Conclusions:**

The HMIS was found to be a unidimensional scale comprised of three conceptual components. There was evidence of good convergent validity, with a medium‐sized correlation between the total moral injury score and burnout. However, correlations were weaker for loss of hope and loss of trust. Moral injury was not significantly associated with loneliness when controlling for mental health difficulties, indicating good divergent validity. Moral injury scores predicted worse severity of depression, anxiety and PTSD, supporting criterion‐related validity. Findings suggest that the HMIS is a valid scale that can be used by researchers to assess moral injury specifically in a health care context.

## INTRODUCTION

Moral injury has been described as the damage caused to an individual's character resulting from acts, whether performed by the individual or witnessed, that are against the person's intrinsic set of ethical values (Griffin et al., 2019; Litz & Maguen, 2012; Shay, 2014). It is generally agreed that moral injury can be decomposed into the potentially morally injurious event, the distress as a result of the event and a sense of betrayal or loss of trust in those who hold legitimate authority (Griffin et al., 2019; Litz & Maguen, 2012; Shay, 2014). Throughout the COVID‐19 pandemic, health care workers, especially front‐line workers, have been exposed to potentially morally injurious events (Borges et al., 2020; Čartolovni et al., 2021; Dean et al., 2020; Ford, 2019; Kopacz et al., 2019; Litam & Balkin, 2021; Roycroft et al., 2020; Williamson et al., 2020). Many health care workers have been forced to make or have witnessed the result of morally challenging decisions, such as allocating resources to those who are most likely to survive (Čartolovni et al., 2021; Roycroft et al., 2020; Williams et al., 2020; Williamson et al., 2020), most likely as a consequence of the scarcity of resources and the increasing demand for patient care (Čartolovni et al., 2021; Williams et al., 2020; Williamson et al., 2020). Faced with morally challenging decisions, health care workers who experience moral injury may feel a loss of trust in both their colleagues and in their employing institution (Čartolovni et al., 2021; Jinkerson, 2016; Shay, 2014), a sense of hopelessness (Čartolovni et al., 2021; Pavlish et al., 2011) and may also experience burnout (Kopacz et al., 2019; Litam & Balkin, 2021; Talbot & Dean, 2018; Williams, 2021; Williamson et al., 2020).

Although moral injury itself is not a mental health disorder (Hall et al., 2021), it is likely to have contributed to the mental health burden observed within health care workers during the recent pandemic (Čartolovni et al., 2021; Danson et al., 2023). For example, Wanigasooriya et al. (2021) reported that greater moral injury is significantly associated with greater severity of anxiety, depression and PTSD. This finding supports previous research demonstrating a link between greater moral injury and adverse mental health in this population (Liberati et al., 2021; Williamson et al., 2020). Based on this evidence, moral injury has been discussed within the literature as one plausible explanation for the rise of mental health difficulties in health care workers during the pandemic (Chirico et al., 2021; Greenberg et al., 2020; Gupta & Sahoo, 2020; Kinman et al., 2020; Magner et al., 2021; Søvold et al., 2021). Hence, moral injury may negatively impact health care workers' everyday emotional, social and behavioural functioning, impairing their ability to function in their roles (Borges et al., 2020; Čartolovni et al., 2021; Litam & Balkin, 2021).

Two standardized measures of moral injury have been employed in research on health care workers: The Moral Injury Events Scale (MIES, Nash et al., 2013) and the Moral Injury Symptoms Scale for Health care Workers (MISS‐HP, Mantri et al., 2020). While both the MIES and the MISS‐HP are valid and reliable measures, there are some conceptual limitations that compromise them as suitable measures for use in the health care systems in secular societies such as the United Kingdom and most European countries. The MIES was designed to assess moral injury in military personnel, not health care workers (Nash et al., 2013). Hence, in addition to contextually inappropriate items (‘perceived betrayal by nonmilitary others’), there are no items that are specific to the possible moral distress experienced by health care workers, such as being unable to adequately provide care to all patients. The MISS‐HP was designed and validated for health care workers living in the USA, particularly in North Carolina. Due to the high percentage of religious individuals living in the state (approximately 80%, Pew Research Centre, 2014), the MISS‐HP embeds religious values into the conceptualisation of moral injury by including such items as: ‘I sometimes feel God is punishing me for what I've done or not done while caring for patients’ and ‘Compared to before I went through these experiences, my religious/spiritual faith has strengthened’. Hence, the MIS reflects, how religious values and teachings have influenced moral values in that particular culture (Atuel et al., 2021).

However, moral injury can occur regardless of an individual's religious or non‐religious beliefs. An individual's moral principles are likely to be developed through their sense of self and wider values, which are learnt through their upbringing and shaped by their social groups, which may not include religious institutions (Atuel et al., 2021). Indeed, there is empirical evidence that, although believers and disbelievers may differ in some aspects of moral reasoning (disbelievers placing more emphasis on consequentialist principles, for example), most people who are atheists can articulate a coherent set of moral beliefs and they are no more likely to evidence characteristics associated with amorality than people who are religious (Ståhl, 2021).

The importance of developing a moral injury measure that does not call on religious concepts is highlighted by sociologists' observation that many countries have experienced a process of secularization or ‘disenchantment’ that began at least as early as the nineteenth century (Bruce, 2003). This process has continued in recent times so that, for example, The World Values survey has reported a sharp global decline between 2007 and 2019 in the number of people who report that God is important in their lives (Inglehart, 2020). This trend has been observed in most countries including the United States (India is the only clear exception) and has been particularly marked in European countries (Cooperman et al., 2017) and especially the United Kingdom where 48.6% of the adult population identified as having ‘no religion’ in 2014 (Bullivant, 2017).

Hence both the MIES and the MISS‐HP appear to have important limitations that are likely to compromise their ability to accurately assess moral injury in health care workers in the United Kingdom and other secularized countries. In the light of these limitations, the current study reports the development of the Health care Moral Injury Scale (HMIS), which was designed to assess moral injury of United Kingdom health care workers. The HMIS conceptualizes moral injury in terms of the loss of hope and trust, as well as the distress that stems from morally injurious events during an individual's occupational duty. Because we chose items in relation to clinical practice and not culture‐specific religious values, we hope that the scale will have applicability beyond the specific health care site and country (an NHS hospital organization in the north of England) in which it was developed and will be useful in a wide range of health care settings. We investigated the validity and reliability of the new scale by testing the following:

1. The items within the HMIS scale will have high internal consistency within the health care sample.
2. The HMIS scale will be unidimensional.
3. The HMIS scale will show good convergent validity with general mistrust, mistrust in authorities, greater burnout and less hope.
4. The HMIS scale will show good divergent validity with loneliness, because loneliness is known to adversely impact mental health (Hards et al., 2022; Loades et al., 2020; Park et al., 2020), but is a theoretically distinct construct from moral injury.
5. The HMIS scale will demonstrate good criterion‐related validity by predicting greater severity of depression, anxiety and PTSD symptoms.

## METHODS

### Participants

Health care workers employed at the Sheffield Teaching Hospital (STH) were invited to take part in multiple survey waves throughout the COVID‐19 pandemic through an invitation emailed from the Trust's Communication Department. Questionnaires were administered online using Qualtrics (2020). The first wave of data was collected between the 2nd and 12th June 2020, towards the end of the first United Kingdom lockdown (national lockdown restrictions ended and local lockdowns were introduced on 29th of June); however, the new moral injury scale was only included in the second and third waves. The second wave of data was collected between 18 September 2020 and 27 October 2020, and included 858 participants who fully completed the survey.

### Measures

#### Moral injury items

We followed recommendations for best practices in constructing and adapting new scales by combining both inductive and deductive approaches to choosing items (Boateng et al., 2018). Specifically, we chose relevant items from the MEIS and adapted them based on clinically‐based authors' observations of events during the early stages of the pandemic. As shown in Table 1, 10 items were chosen and adapted to reflect moral injury defined as the distress, weariness and loss of trust that stems from the internal conflict between witnessing or performing acts of transgression, and the individual's intrinsic ethical values. Respondents are asked to state the extent to which they agree or disagree with statements such as ‘I have felt let down by leaders in [institution] who I previously trusted’ and ‘I have been unable provide the care I usually aspire to’. In this case, the institution was STH which was the employer of the participating health care workers. Yet, the scale could be potentially used in other organizations by substituting the appropriate name. Responses were rated on a five‐point Likert scale, with higher scores reflecting greater moral injury (1 = ‘Strongly disagree’, 5 = ‘Strongly agree’).

**TABLE 1:** Summary of the means and standard deviations for the HMIS item.

Statements	M	SD
1. I have felt let down by leaders in [Institution] who I previously trusted.	2.95	1.28
2. I have been unable to provide the care I usually aspire to.	2.95	1.20
3. I have had to act in ways that are inconsistent with my moral code or values.	2.30	1.21
4. I felt let down by fellow health care workers who I previously trusted.	2.45	1.26
5. I have been unable to do all the things I should have done.	3.10	2.22
6. I have been distressed by witnessing patients not receiving the care they should have received.	2.68	1.28
7. I have been forced to deal with situations that no one in my position should have to face.	2.34	1.24
8. I have been distressed by the need to make morally troubling decisions.	2.20	1.16
9. I have been troubled by witnessing acts that are inconsistent with my moral code or values.	2.10	1.16
10. I felt let down by NHS leaders outside [Institution] who I previously trusted.	2.79	1.26

*Note*: Question: Please indicate how much you agree or disagree with each of the following statements regarding your experiences of working at [Institution] during the coronavirus pandemic. [Institution] = For this sample, the institution was the Sheffield Teaching Hospital (STH).

#### Measures to test convergent validity

##### General mistrust

General mistrust was assessed by a single item asking the respondents: ‘Generally speaking, would you say that most people can be trusted or that you need to be very careful in dealing with people?’(adapted from: Tranter & Skrbiš, 2009). Responses were rated on a 5‐point Likert Scale, with, higher scores reflecting greater mistrust (1 = ‘Most people can be trusted’, 5 = ‘Need to be very careful’).

##### Trust in authorities

Trust in authorities was assessed using an 8‐item scale developed for the COVID‐19 Psychological Research Consortium longitudinal study of mental health in the UK population during the pandemic (McBride et al., 2021). Respondents were asked: ‘Could you indicate how much trust you have in the following institutions’, followed by ‘Parliament’, ‘The government’, ‘The police’, ‘The legal system’, ‘Political parties’, ‘Scientists’, ‘Doctors and other health professionals’, and ‘The Department of Health and Social Scare’. Responses were rated on a 5‐point Likert scale, with higher scores reflecting greater mistrust (1 = ‘Completely trust’, 5 = ‘Do no trust at all’). The scores for each item are summed together to create a total score for mistrust in authorities. In the health care sample, the alpha coefficient for the eight items was high, .83, indicating good internal consistency.

##### Hope

Hope was assessed by a single item taken from the Hopelessness Scale (Everson et al., 1996). The item asks the respondent to indicate the extent to which they agree or disagree with the following statement: ‘The future seems to me to be hopeful, and I believe that things are changing for the better’. Responses are rated on a 5‐point Likert scale with higher scores reflecting greater hope (1 = ‘Absolutely disagree’, 5 = ‘Absolutely agree’).

##### Burnout

The 16‐item Oldenburg Burnout Inventory (OBI, Demerouti & Bakker, 2008) was used to assess burnout. The inventory contains two subscales, with 8 items each, assessing exhaustion and disengagement (Demerouti & Bakker, 2008). Items from the exhaustion subscale include ‘When I work, I feel energised’, and items from the disengagement subscale include ‘I always find new and interesting aspects of my work’. Items are rated on a 4‐point Likert scale, and once appropriate items are reversed, higher scores indicate greater burnout (1 = ‘Strongly disagree’, 4 = ‘Strongly agree’). Once the appropriate items are reversed, the scores are summed together to create a total burnout score. The OBI has demonstrated good internal consistency in previous studies (for both subscales: alpha = .85) (Demerouti & Bakker, 2008) and in the current study (for both subscales: alpha = .83).

#### Measures to test divergent validity

##### Loneliness

Loneliness was assessed by a single item ‘During the past week, have you felt lonely?’ (adapted from: Russell et al., 1980). Items are rated on a 4‐point Likert scale, with higher scores indicating greater loneliness (1 = ‘Rarely or none of the time’, 4 = ‘All of the time’).

#### Measures to test criterion‐related validity

##### Depression

Depression was assessed through the 9‐item Patient Health Questionnaire (PHQ‐9) (Kroenke et al., 2001), a reliable, valid and widely adopted measure that assesses symptom severity. Items include ‘Over the last 2 weeks, how often have you been bothered by the following problems? Little interest or pleasure in doing things?’ and ‘Feeling down, depressed, or hopeless?’. Items were rated on a 4‐point Likert scale, with higher scores indicating greater severity of depression (0 = ‘Not at all’, 3 = ‘Nearly every day’). Scores from each item are summed together to create a total score reflecting the severity of depression. A categorical variable can also be created to indicate the probable diagnosis of depression, whereby a total score greater than or equal to 10 indicates a probable diagnosis. The PHQ‐9 has demonstrated good internal consistency in previous research (alphas from .87 to .89) (Beard et al., 2016; Kocalevent et al., 2013; Shin et al., 2019) and in the current study (alpha = .89).

##### Anxiety

Anxiety was assessed with the 7‐item General Anxiety Disorder scale (GAD‐7) (Spitzer et al., 2006), a reliable, valid and widely adopted measure. Items include ‘Over the last 2 weeks, how often have you been bothered by the following problems? Feeling nervous, anxious or on edge?’ and ‘Not being able to stop or control worrying?’. Items were rated on a 4‐point Likert scale (0 = ‘Not at all’, 3 = ‘Nearly every day’). A total score of the GAD‐7 indicates the severity of anxiety, with a total score of greater than or equal to 10 indicating a probable diagnosis. The GAD‐7 has demonstrated good internal consistency in previous research (alpha = .88) (Beard & Björgvinsson, 2014; Johnson et al., 2019) and in the current study (alpha = .92).

##### Post‐traumatic stress disorder

Symptoms of PTSD were assessed using the 9‐item International Trauma Questionnaire (ITQ) (Cloitre et al., 2018), a reliable and valid screening measure for assessing severity of PTSD symptoms. Health care workers were asked to respond to the items in relation to the recent COVID‐19 pandemic. Items include ‘Being “super‐alert”, watchful, or on guard?’ and ‘Feelings jumpy or easily startled?’. The ITQ includes three subscales, each with three items, measuring clusters of trauma symptoms (‘avoidance’, ‘sense of threat’, ‘re‐experiencing in the here and now’) and three items assessing the individual's level of functioning. The total score of the ITQ is the sum of all the items from the three symptom clusters only. Items are rated on a 4‐point Likert scale with higher scores indicating greater severity of PTSD (0 = ‘Not at all’, 4 = ‘Extremely’). A score greater than or equal to two for all three clusters as well as at least one of the function items (as defined as a score greater than or equal to two) indicates a probable diagnosis of PTSD. The ITQ has demonstrated good internal consistency in previous studies (alpha = .89) (Cloitre et al., 2021) and in the current study (alpha = .91).

### Statistical analysis

All analysis was performed using SPSS‐27 (IBM Corp., Released 2020) apart from the Confirmatory Factor Analysis for which AMOS (Arbuckle, 2019) was used. As recommended by DeVellis and Thorpe (2021), the authors identified the most likely factor structure of the HMIS by applying their theoretical understanding of moral injury, as informed by the existing literature and then used statistical analyses to test the properties of the scale to ensure its usefulness in future studies. This approach was adopted because widely used moral injury measures have been criticized for overly relying on face validity (Houle et al., 2024; Litz & Kerig, 2019; McEwen et al., 2021). To determine the most likely factor structure of the HMIS, we used the replication approach suggested by DeVellis and Thorpe (2021). The sample was therefore split at random to produce two independent samples to test and confirm the factor structure. Exploratory Factor Analysis (EFA) was performed on one sub‐sample and then Confirmatory Factor Analysis (CFA) was conducted with the second.

Although the data were not normally distributed, maximum likelihood estimation was used to estimate both EFA and CFA models because the sample sizes were considered adequate and the deviation was not considered severe. An oblique (oblimin) rotation was employed for the EFA.

Although the EFA results suggested a one‐factor solution, the CFA compared three models to confirm the factor structure of the HMIS against other plausible factor structures pertaining to current theoretical concepts of moral injury (Griffin et al., 2019; Litz & Maguen, 2012; Shay, 2014) and the semantic similarities between items. These were a unidimensional model, a correlated two‐factor model (‘Event’ and ‘Distress’) and a correlated three‐factor model (‘Event’, ‘Distress’ and ‘Loss of trust’). As there is currently no best method for comparing CFA models, it is generally recommended that Akaike Information Criterion (AIC) and Bayesian Information Criterion (BIC) are used for model selection, where a lower value indicates a better fitting model. However, the Chi‐Square test, Root Mean Square Error of Approximation (RMSEA) were all calculated to evaluate the fit of the models (Greiff & Scherer, 2018; Kuha, 2004; Vrieze, 2012; Xia & Yang, 2019).

As the results from the EFA and the CFA found the factor scale to be unidimensional with three conceptual components (‘Event’, ‘Distress’ and ‘Loss of trust’), items were summed together to generate a total score. Cronbach's alpha was calculated to determine the internal consistency of the 10 items of the HMIS for the whole sample. Further analyses were performed to determine if the HMIS has good reliability for front‐line (nurses, doctors, therapists) and non‐front‐line workers (administration, domestic and, scientific and technical staff). CFA was used to test configural and metric factorial invariance of the HMIS to determine if the factor structure is the same for front‐line workers and non‐front‐line workers.

Correlational analyses were conducted to determine the convergent and divergent validity of the HMIS, and to provide preliminary evidence of its nomological network. For convergent validity, correlations (Pearson's *r*) were calculated to determine the association between the HMIS and hope, burnout (exhaustion and disengagement), general mistrust and mistrust in institutions. As a guide, the constructs are assumed to be related if they reveal a medium to large correlation (above .30) (Robinson, 2018). For divergent validity, a Pearson's *r* was calculated to determine the association between the HMIS and loneliness. It was expected that the HMIS would not be strongly associated with loneliness, because moral injury is a distinct and unrelated construct. As a guide, the constructs are considered unrelated if they reveal a low association (below .20) (Robinson, 2018). Multiple linear regressions were conducted to determine the criterion‐related validity of the HMIS, and specifically to determine if, as predicted, greater moral injury was significantly associated with higher levels of depression, anxiety and PTSD.

## RESULTS

### Descriptive analyses

From the second survey wave, 858 completed all 10 items of the HMIS. The mean age of the sample was 44.56 (SD = 11.87; Range = 18–72). Most of the sample were females at 81%, (*n* = 693), 18% were males (*n* = 158), the rest either reported that they were transgender, preferred not to say or were described as ‘other’ (*n* = 7). The observed gender ratio was expected due to the known percentage of female HCWs in the STH and the United Kingdom (77%) (NHS Digital, 2019). The largest occupational group were administrative and managerial staff at 33% (*n* = 285), 30% were nurses (*n* = 254), 8% were doctors (*n* = 67), 8% were scientific and technical staff (*n* = 66), 7% were therapists (*n* = 61), 1% were domestic or porting staff (*n* = 5) and lastly, 14% reported that they were ‘other’ (*n* = 116). The sample was predominately white British at 92% (*n* = 791); other ethnic groups, including minority groups, accounted for 8% of the sample (*n* = 61). The mean score of the HMIS was 25.90 (SD = 8.47; Range = 10–50). Table 1 provides the details, means and standard deviations of each item.

### Confirming the internal structure of the scale

**FIGURE 1:**
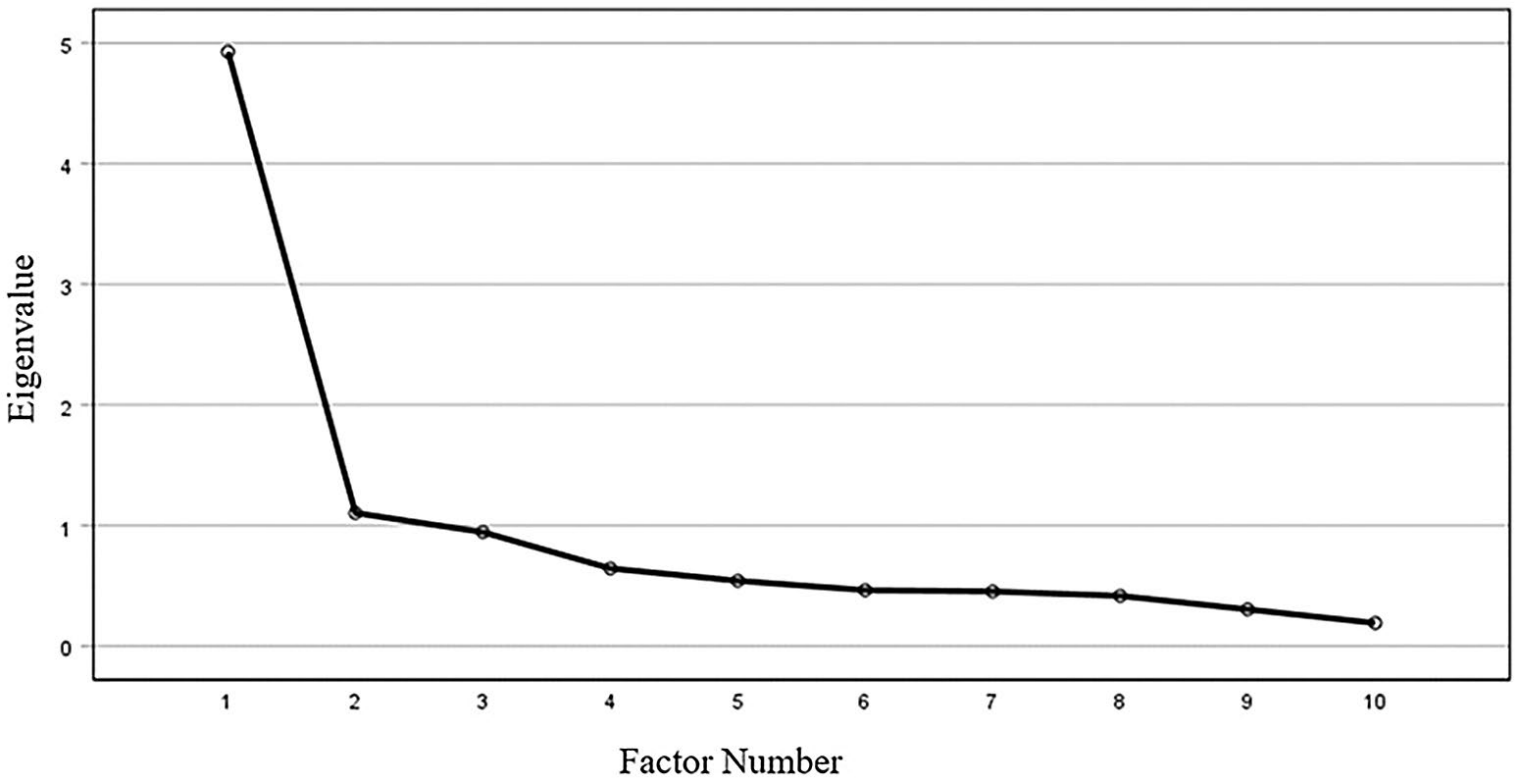
Scree plot from the first Exploratory Factor Analysis for the HMIS (*N* = 406).

When EFA was performed on the first randomly split sub‐sample (*n* = 406), the results from the Scree plot of the eigenvalues (Figure 1) and the factor loadings (Table 2.) suggested that a one‐factor solution was viable, as indicated by an observable drop in eigenvalues after the first factor (Cattell, 1966). However, it should be noted that the eigenvalues for the second and third factors were still somewhat high and just below 1.

**TABLE 2:** Factor loadings for the HMIS items.

HMIS items	Factor loadings		
	One‐factor solution	Two‐factor solution	
		1	2
1. I have felt let down by leaders in [Institution] who I previously trusted	.54	.83	.15
10. I felt let down by NHS leaders outside [Institution] who I previously trusted	.63	.62	−.12
4. I felt let down by fellow health care workers who I previously trusted	.55	.64	−.01
3. I have had to act in ways that are inconsistent with my moral code or values	.69	.45	−.31
2. I have been unable to provide the care I usually aspire to	.49	.38	−.15
5. I have been unable to do all the things I should have done	.51	.38	−.18
8. I have been distressed by the need to make morally troubling decisions	.84	−.11	−.99
9. I have been troubled by witnessing acts that are inconsistent with my moral code or values	.84	.07	−.81
7. I have been forced to deal with situations that no one in my position should have to face.	.80	.24	−.60
6. I have been distressed by witnessing patients not receiving the care they should have received.	.60	.22	−.40

*Note*: [Institution] = For this sample, it was the Sheffield Teaching Hospital (STH).

**FIGURE 2:**
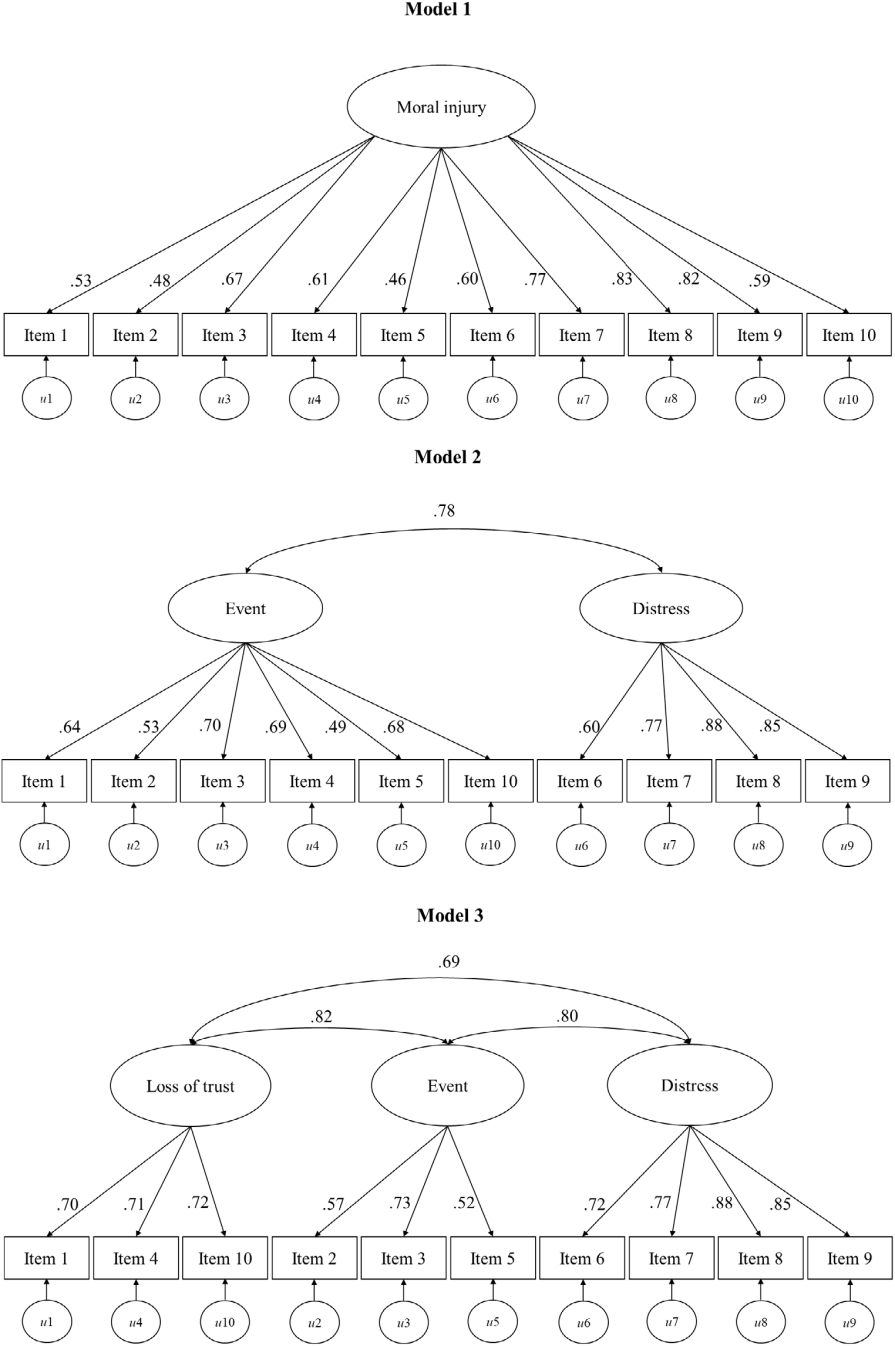
CFA and the standardized estimates of all three models.

Confirmatory Factor Analysis (CFA) tested three models (see Figure 2.) using the second sub‐ sample (*n* = 427). First, we considered a unidimensional model. Secondly, a correlated two‐factor model was evaluated with items assigned to factors corresponding to events and their consequences based on the results of the EFA (see Table 2.) and the theoretical distinction between PMIEs and the distress they provoke (McEwen et al., 2021). Finally, and despite the results of the EFA, a correlated three‐factor model was also estimated with items assigned to potential factors corresponding to loss of trust, the event (PMIE) and the distress (as a result of the PMIE). We considered this model because of the similarities in the wording of some of the items (i.e., ‘felt let down… who I previously trusted’), and because ‘loss of trust’, PMIE and distress as a result of PMIE are considered to be a core component of moral injury by some scholars (Čartolovni et al., 2021; Jinkerson, 2016; Williamson et al., 2020). Regarding semantic similarities, the three items 1, 10 and 4 within the HMIS were worded to reflect feelings of loss of trust; the three items 3, 2, 5 were worded to refer to exposure to potentially morally injury events (PMIE) that are likely to occur specifically within a health care context; and finally, the items 8, 9, 7 and 6 were worded to reflect to the experience of distress due to the exposure of a PMIE. An additional consideration was that separate alpha coefficients for each moral injury component were acceptable (for loss of trust, alpha = .74; for event, alpha = .73; for distress alpha = .82). Therefore, although the EFA results suggests a one or two‐factor solution, for the Confirmatory Factor Analysis (CFA) a three‐factor model was also evaluated.

The model fit indices (Table 3.) demonstrate that the correlated three‐factors model (model 3) was most suitable, suggesting that the HMIS is likely to map onto three conceptual factors: the event, the distress following the event and loss of trust for the institution. However, the correlations between the three conceptual factors were high (.82, .80 and .69; see Figure 2), which was unsurprising as these are key components of moral injury (Čartolovni et al., 2021; Jinkerson, 2016; Williamson et al., 2020). These correlations might suggest the existence of a general factor that underpins the three subordinate factors or conceptual components of moral injury. Therefore, based on the model fit indices, our current understanding of moral injury, the HMIS was found to be a unidimensional scale with three key conceptual components (the event, the distress following the event and loss of trust for the institution). Therefore, although it is possible to use three subscales to measure the three components separately, it is recommended that a total moral injury score is used for most purposes, and this was the approach we took for the rest of the investigation.

**TABLE 3:** Summary of the model fit indices for the three CFA models.

Model	*χ* ^2^ (*df*)	RMSEA	TLI	CFI	AIC	BIC
Unidimensional	*χ* ^2^ (35) = 307.50***	.14	.81	.85	347.50	428.64
Correlated two‐factor model	*χ* ^2^ (34) = 181.83***	.10	.89	.92	223.83	309.03
Correlated three‐factor model	*χ* ^2^ (32) = 149.58***	.09	.91	.94	195.58	288.88

*Note:* ***p < .001.

### Reliability

Using a moral injury total score, the HMIS scale demonstrated good internal consistency (alpha = .88). Using model 3 (see Figure 2), the internal consistency and factorial (configural and metric) invariance were also tested for front‐line and non‐front‐line workers because moral injury may be viewed and experienced differently by health care workers in each of these roles. Specifically, items such as ‘I have been unable to provide the care I usually aspire to’ and ‘I have been distressed by witnessing patients not receiving the care they should have received’ may not be as relevant for the non‐front‐line workers. Cronbach's alpha revealed good to acceptable internal consistency in the HMIS for both the front‐line workers (total score, alpha = .87; loss of trust, alpha = .73; event, alpha = .69; distress, alpha = .86) and non‐front‐line workers (total score, alpha = .88; loss of trust, alpha = .73; event, alpha = .69; distress, alpha = .86). Configural invariance (same model) through CFA (using model 3; see Figure 2) was performed revealing an acceptable model fit (*χ* ^2^ (64) = 261.92, *p* < .001; RMSEA = .07; TLI = .91; CFI = .94; AIC = 353.92; BIC = 356.76). To test the metric invariance (equal loadings) a chi‐squared test was performed between the unconstrained model and the measurement weighted model revealing no significant difference (*χ* ^2^ (7) = 6.05, *p* = .53). Together, both the configural and metric invariance tests suggest that the HMIS is likely to have the same factor structure (model 3) for front‐line workers and non‐front‐line workers demonstrating high reliability across the groups.

Although the data violated the assumption of normality, the deviation was not considered severe and therefore a *t*‐test was performed to compare moral injury scores between front‐line workers and non‐front‐line workers, revealing that front‐line workers had significantly higher moral injury mean scores (M = 27.26, SD = 8.57) compared to non‐front‐line workers (M = 24.91, SD = 8.11), (*t*(736) = 3.83, *p* < .001). This difference was expected because front‐line workers have more direct exposure to morally injurious situations.

### Testing the convergent and divergent validity of the HMIS

HMIS scores were significantly associated with higher general mistrust (*r* = .22, *p* < .001), higher mistrust in authorities (*r* = .26, *p* < .001), lower hope (*r* = −.21, *p* < .001) and higher burnout (exhaustion, *r* = −.41, *p* < .001; disengagement, *r* = −.38, *p* < .001). This pattern of findings provides support for the convergent validity of the scale. The test of divergent validity found that the HMIS was, unexpectedly, significantly associated with loneliness (*r* = .26, *p* < .001). A plausible explanation of this unexpected result could be that loneliness and mental health difficulties (depression, anxiety and PTSD) are often highly correlated (Hards et al., 2022; Loades et al., 2020; Park et al., 2020). Therefore, further post hoc testing was performed using a partial correlation (2‐tailed) to control for depression (PHQ9 total score), anxiety (GAD7 total score) and PTSD (ITQ total score). This revealed that, after controlling for depression, anxiety and PTSD, the HMIS score was no longer significantly associated with loneliness (*r* = .06, *p* = .12), indicating that the HMIS demonstrates good divergent validity with loneliness.

### Testing the criterion‐related validity of the HMIS

Three linear regressions were performed to determine if higher moral injury scores predicted higher PHQ9, GAD7 and ITQ scores. The model for PHQ9 was significant (*n* = 879, *F*(1,847) = 120.59, *p* < .001), with a *R*‐square of .13(unstandardised B = .25, standardized *β* = .35, *p* < .001). The model for GAD7 was also significant = (*n* = 845, *F*(1,843) = 107.06, *p* < .001), with a *R*‐square of .11 (unstandardised B = .22, standardized *β* = .34, *p* < .001). Finally, the model for the ITQ was significant (*n* = 818, *F*(1,816) = 128.03, *p* < .001), with a *R*‐square of .14 (unstandardised B = .21, standardized *β* = .37, *p* < .001). Consistent with our hypotheses, the HMIS predicted greater severity of depression, anxiety and PTSD in health care workers.

## DISCUSSION

The aim of the current study was to introduce and test the construct validity of a new measure of health care moral injury: the HMIS. Factor analysis led us to find that the HMIS is likely an unidimensional scale measuring moral injury in health care workers, but can be decomposed into three key conceptual components: PMIEs, the distress resulting from the PMIEs and a loss of trust. The tests of construct validity suggest that moral injury in health care workers is related but distinct from general mistrust, mistrust in authorities, hope, burnout and disengagement. When controlling for mental health difficulties, there was no association with loneliness, which suggests good divergent validity. The HMIS also demonstrated good criterion‐related validity by being associated with greater severity of depression, anxiety, and PTSD. It is also worth noting that HMIS scores were higher in front‐line health care workers than in non‐front‐line workers, which could be taken as additional evidence of the validity of the scale. Together, these findings provide support for the convergent, divergent and criterion‐related validity of the HMIS, and help to establish the nomological network of moral injury as measured by the HMIS.

Our findings suggest that the HMIS is a useful instrument across diverse health care occupational roles. The importance as well as a strength of this finding is that, compared to the MISS‐HP (Mantri et al., 2020), the HMIS is perhaps more able to measure moral injury across health care occupational roles, including nurses and non‐medical staff. Therefore, HMIS could be considered a suitable measure when understanding moral injury in a health care sample consisting of front‐line and non‐front‐line staff. Additionally, the current investigation supports the findings by Williamson et al. (2020) which revealed that there is likely a significant difference in levels of moral injury between front‐line and non‐front‐line workers. A higher moral injury score is unsurprising as front‐line workers are exposed to more morally injurious events compared to non‐front‐line workers, as they provide direct care to patients. This suggests that front‐line workers may be more vulnerable to the consequences of moral injury, such as an increase in severity of depression, anxiety and PTSD, which the literature supports (Williamson et al., 2020).

The results of the convergent and divergent validity tests of the HMIS replicate and extend our understanding of moral injury as experienced in a health care context. Burnout was used to test the convergent validity of the MISS‐HP (Mantri et al., 2020) and a medium‐sized correlation was also found in that study. The current investigation used the Oldenburg Burnout Inventory (Demerouti & Bakker, 2008), whereas Mantri et al.'s study used the Maslach Burnout Inventory (Maslach et al., 1997). However, both measures assume that emotional exhaustion is a key element of burnout. Hence, the findings from both studies provide support for the notion that moral injury is a related, but separate, construct to burnout or more specifically emotional exhaustion.

However, in contrast to our findings for burnout, the associations of moral injury between loss of hope, and between loss of trust, were modest. Loss of trust and hope have not previously been tested as measures for establishing convergent validity despite their importance for moral injury (Čartolovni et al., 2021; Jinkerson, 2016; Kopacz et al., 2019; Litam & Balkin, 2021; Pavlish et al., 2011; Shay, 2014; Talbot & Dean, 2018; Williams, 2021; Williams et al., 2020). One possible explanation is that moral injury is a complex concept and that, in addition to loss of hope and trust, likely includes, feelings of guilt, shame, betrayal, as well as a loss of meaning or purpose (Koenig et al., 2018). The nomological network of moral injury may include many related, but distinct, constructs and it is likely that these include loss of hope and trust. As noted previously, while the HMIS assesses moral injury as a unidimensional construct, it is important to consider the complexity of moral injury as a concept (McEwen et al., 2021; Shay, 2014), and this includes the notion of hope and trust within the health care setting.

Initially, we were unable to establish divergent validity for the HMIS with respect to loneliness because, unexpectedly, loneliness was significantly associated with moral injury. Recent studies have found that moral injury in health care workers may lead to disruptions in relationships with family and friends. However, this is most likely due to the loss of trust experienced (Mantri et al., 2020; Nash et al., 2013; Shay, 2012, 2014), rather than because of an association with loneliness. Further post hoc testing was performed to control for mental health difficulties, as these are often highly correlated with loneliness (Hards et al., 2022; Loades et al., 2020; Park et al., 2020). Findings revealed that, after controlling for mental health difficulties, MI as measured by the HMIS, is a distinct and unrelated concept to loneliness. This demonstrates that the HMIS has good divergent validity.

The tests of criterion‐related validity support our hypotheses that moral injury as assessed by the HMIS predicts greater severity of depression, anxiety and PTSD in health care workers. This is consistent with the large and emerging body of literature around the burden on health care workers during the recent COVID‐19 pandemic (Čartolovni et al., 2021; Litam & Balkin, 2021; Sirois & Owens, 2021; Williams et al., 2020). There is a need to understand and support those likely to suffer from moral injury to reduce or counteract the negative mental health impact (Čartolovni et al., 2021; Litam & Balkin, 2021; Talbot & Dean, 2018; Williams et al., 2020). Even before COVID, there had been discussion about the impact of moral injury on health care workers and the possibility that it contributes to mental health difficulties in this population (Talbot & Dean, 2018). It is unsurprising therefore that higher moral injury scores, as assessed by the HMIS, predicted greater severity of adverse mental health outcomes in the current study. Further research is needed to understand the relationship more fully between moral injury and mental health and test interventions that can reduce its effect on health care workers during health emergencies.

### Strengths and limitations

There are several limitations and strengths that need to be considered when interpreting the findings from the current study. The main limitation is that STH is a large organization employing approximately 17,000 staff, yet only 858 completed the survey containing the HMIS. It is possible that those either less or more likely to experience moral injury were most likely to participate in the study. However, this should not compromise the evaluation of the construct validity of the HMIS, as a sufficiently large sample was recruited. Additionally, the HMIS was administered during the COVID‐19 pandemic where the exposure of PMIE was likely higher than usual. During the pandemic, health care workers were especially likely to witness morally challenging events requiring them to make morally challenging decisions (Čartolovni et al., 2021; Roycroft et al., 2020; Williams et al., 2020; Williamson et al., 2020). Hence, the total mean score as presented in the current investigation is likely higher than if the HMIS had been administered outside of a pandemic. Caution is therefore required when comparing mean scores of moral injury, as assessed by the HMIS, across different time periods or situations. Beyond this, however, the validation of the HMIS during the COVID‐19 pandemic could also be viewed as a strength, as it was conducted in a highly relevant context for understanding the correlates and impact of moral injury. Therefore, the HMIS is likely to be suitable for the assessment of moral injury to health care workers during future health crises.

The HMIS has some clear advantages over comparable scales. It builds on our current understanding of moral injury by introducing a health care‐focused scale without the inclusion of religious beliefs. The HMIS appears to be a good measure of moral injury for all health care workers, regardless of their belief systems, which is particularly important in the UK, which is a secular society (Bullivant, 2017). It would be interesting, in a future study, to examine whether the HMIS is associated with the MISS‐HP in subsamples of religiously devout health care workers. Additionally, a strength of the HMIS is that, alongside deductive methods, inductive methods were adopted during its design. Some of the authors were health care workers who worked throughout the pandemic. Items were informed based on these authors' experience and observations of moral injury during their clinical practice. This means that the items were not only designed based upon our current theoretical understanding of moral injury (deductive), but also through first‐hand knowledge imparted by health care workers themselves (inductive). Integration of inductive methods ensures that the phenomenon is understood through a real‐world perspective and thus, is more accurately represented than by deductive methods alone (O'Neill & Sevastos, 2013; Spector, 2017).

## CONCLUSION

The present investigation provides preliminary evidence that the HMIS is a valid and acceptable measure of moral injury as experienced by health care workers. Consistent with previous theory and research, moral injury, as assessed by the HMIS, predicts greater severity of depression, anxiety and PTSD. The current findings indicate that HMIS may be a useful tool for future investigations of the moral injury experienced by health care workers and its effects on mental health.

## AUTHOR CONTRIBUTIONS

**Kathryn Fradley:** Investigation; writing – original draft; formal analysis; visualization. **Fuschia Sirois:** Conceptualization; investigation; writing – review and editing; supervision; validation; methodology; data curation; funding acquisition. **Richard Bentall:** Conceptualization; investigation; funding acquisition; methodology; validation; writing – review and editing; supervision; data curation. **Jaydip Ray:** Data curation; writing – review and editing; conceptualization; methodology. **Rhian Bishop:** Data curation; funding acquisition; project administration; resources; writing – review and editing; conceptualization; methodology. **Jonathan Wadsley:** Conceptualization; data curation; writing – review and editing; methodology. **Sarah Danson:** Conceptualization; investigation; funding acquisition; writing – review and editing; validation; project administration; resources; supervision; data curation; methodology.

## PATIENT AND PUBLIC INVOLVEMENT

The current study was conducted and performed in collaboration between psychological researchers at the Sheffield Teaching Hospital. Beyond the authors, no other NHS staff were involved in the planning or execution of the current study.

## Data Availability

The data that support the findings of this study are available from the corresponding author upon reasonable request.
